# Supplementary material for: Causal Relationship between Aging and Anorexia Nervosa: A White-Matter-Microstructure-Mediated Mendelian Randomization Analysis
Source: Biomedicines. 2024 Aug 16;12(8):1874. doi: 10.3390/biomedicines12081874 (PMC11351342; doi:10.3390/biomedicines12081874)
Supplement: Supplementary file 1 [file biomedicines-12-01874-s001.zip › Supplementary Figure/Figure S8/Leaveoneout plot of MR analysis for TL to ubm-b-1559.pdf]

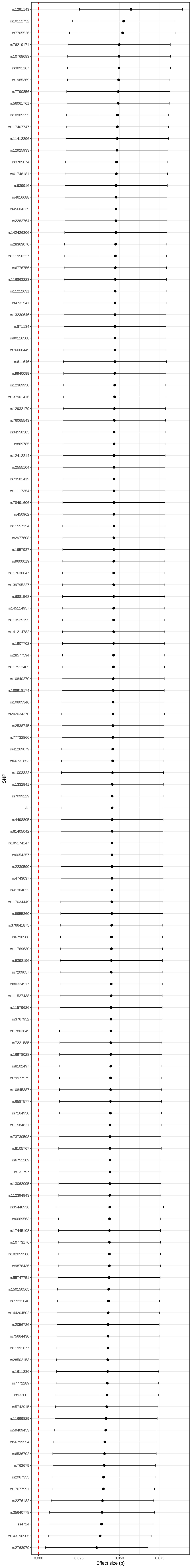
SNP
rs1291143
rs10112752
rs7705526
rs76219171
rs10768683
rs3891167
rs1985369
rs7790856
rs56061761
rs10905255
rs117407747
rs11412296
rs12925933
rs3785074
rs61748181
rs939916
rs4616688
rs45604339
rs2282764
rs142426306
rs28363070
rs111950327
rs6776756
rs116863223
rs11212631
rs4731541
rs13230646
rs871134
rs80116508
rs76666449
rs611646
rs9940099
rs12369950
rs137901416
rs12932179
rs76065543
rs34550383
rs869785
rs12412214
rs2555104
rs73581419
rs11117354
rs78491606
rs450962
rs11557154
rs2977608
rs1957937
rs9600019
rs117630647
rs139795227
rs6881568
rs145114957
rs113525195
rs141214782
rs1907702
rs28577594
rs117512405
rs10840270
rs188918174
rs10805346
rs202034370
rs2538745
rs77732866
rs41269079
rs66731853
rs1003322
rs1332941
rs7099229
A8
rs4498805
rs61405042
rs185174247
rs6054257
rs2230590
rs4743037
rs41304832
rs117034449
rs9955360
rs376641875
rs6790988
rs11769630
rs9398196
rs7209057
rs80324517
rs111527438
rs11579626
rs3767952
rs17803849
rs7221585
rs16978028
rs8102497
rs79977579
rs10845387
rs6587577
rs7164950
rs11584821
rs73730598
rs8105767
rs6751209
rs131797
rs13062095
rs112394943
rs35446936
rs6669563
rs17445108
rs10773176
rs182059586
rs9878436
rs55747751
rs150150565
rs77231040
rs144204502
rs2056726
rs75664430
rs11991877
rs28502153
rs1611236
rs7772289
rs932002
rs5742915
rs11699829
rs59409453
rs56799554
rs6536702
rs762679
rs2967355
rs17677991
rs2276182
rs35640778
rs4724
rs143190905
rs2763979
0.000
0.025
0.050
0.075
Effect size (b)
